# Tacrolimus ameliorates tubulointerstitial inflammation in diabetic nephropathy via inhibiting the NFATc1/TRPC6 pathway

**DOI:** 10.1111/jcmm.15562

**Published:** 2020-08-11

**Authors:** Shumin Zhang, Huafen Wang, Yifei Liu, Wenxia Yang, Jialu Liu, Yuzhang Han, Yu Liu, Fuyou Liu, Lin Sun, Li Xiao

**Affiliations:** ^1^ Department of Nephrology The Second Xiangya Hospital Central South University Changsha China

**Keywords:** diabetic nephropathy, inflammation, NFATc1, tacrolimus, TRPC6, tubular cell

## Abstract

Tubulointerstitial inflammation is crucial for the progression of diabetic nephropathy (DN), and tubular cells act as a driving force in the inflammatory cascade. Emerging data suggested that tacrolimus (TAC) ameliorates podocyte injury and macrophage infiltration in streptozotocin (STZ) mice. However, the effect of TAC on tubulointerstitial inflammation remains unknown. We found that albuminuria and tubulointerstitial damage improved in db/db mice treated with TAC. Macrophage infiltration and expression of IL‐6, TNF‐α, fibronectin, collagen 1 and cleaved caspase 3 were inhibited as well. In addition, the expression of nuclear factor of activated T cell 1 (NFATc1) and transient receptor potential channel 6 (TRPC6) was up‐regulated in the kidneys of DN patients and correlated with tubular injury and inflammation. The expression of NFATc1 and TRPC6 also increased in the kidneys of db/db mice and HK‐2 cells with high glucose (HG), while TAC inhibited these effects. HG‐induced inflammatory markers and apoptosis were reversed by TAC and NFATc1 siRNA in HK‐2 cells, which was abolished by TRPC6 plasmid. Furthermore, HG‐induced TRPC6 expression was inhibited by NFATc1 siRNA, while NFATc1 nuclear translocation was inhibited by TAC, but was restored by TRPC6 plasmid in HK‐2 cells under HG conditions. These findings suggest that TAC ameliorates tubulointerstitial inflammation in DN through NFATc1/TRPC6 feedback loop.

## 1 INTRODUCTION

Diabetic nephropathy (DN) is one of the most serious microvascular complications of diabetes mellitus and is now the leading cause of end‐stage renal disease (ESRD) worldwide.^1^ DN is traditionally thought to be a non‐immune disease that is mainly mediated by metabolic abnormalities and haemodynamic factors.^2^, ^3^ Unfortunately, current strategies including precise control of blood glucose, hyperlipidaemia and pressure still do not effectively delay the progression of DN.^4^, ^5^ Accumulating evidence suggests that inflammation plays a crucial role in the pathogenesis and progression of DN, which is characterized by increased macrophage infiltration and release of multiple inflammatory cytokines.^6^, ^7^, ^8^ Moreover, it was demonstrated that renal tubular epithelial cells (TECs) are a key driving force in mediating macrophage recruitment and the subsequent local inflammatory cascade in the kidney under a hyperglycaemic state.^9^, ^10^, ^11^ Thus, targeting TEC‐mediated inflammation might be a novel therapeutic strategy to alleviate activation of macrophages and kidney inflammation, eventually delaying the progression of DN.

Tacrolimus (TAC, also known as FK506), a new immunosuppressive drug, has lower nephrotoxicity than cyclosporine, which has been widely used in post–kidney transplantation and immune‐related chronic kidney disease by suppression of nuclear factor of activated T cell (NFAT) dephosphorylation and transcriptional activation of NFAT target genes.^12^, ^13^, ^14^ Recently, TAC was attempted to be used in experimental DN patients and animal models. JIN H et al reported that TAC combined with a double dose of valsartan treatment ameliorated proteinuria and improved renal function by inhibiting high‐sensitivity C‐reactive protein and adiponectin levels in a small sample of DN patients.^15^ Qi XM found that treatment with TAC mitigated collagen IV and TGF‐β1 expression in streptozotocin (STZ)‐induced diabetic rats.^16^ Subsequent studies focused on the protective effect of TAC on podocytes and showed restored nephrin and podocin expression in early DN rats.^17^ Regarding inflammation, it was shown that TAC reduced the infiltration of iNOS^+^/ED‐1^+^ macrophages and NF‐кB expression in STZ‐induced diabetic rats.^18^ However, the role of TAC in tubular‐induced inflammation remains unknown.

The canonical transient receptor potential channel 6 (TRPC6) is a subtype of non‐selective Ca^2+^‐permeable cation channels.^19^ It was shown that TRPC6 expression was up‐regulated in the kidneys of STZ rats and Akita mice.^20^, ^21^ TRPC6 mediated podocyte calcium influx enhancement and renal damage in STZ rats.^20^ Emerging evidence demonstrated TRPC6 contributes to inflammation through correlation with NFAT. It was shown that TAC ameliorates podocyte injury in type 2 DN rats by down‐regulating TRPC6 and NFAT expression.^22^ Angiotensin II (Ang Ⅱ) increased TRPC6 expression via an NFAT positive feedback pathway,^23^ whereas the correlation between TRPC6 and NFAT in the inflammation of tubular cells under DN conditions is unclear.

In this study, we focused on the effect of TAC on tubulointerstitial inflammation and injury under a DN state in vitro and in vivo. NFATc1/TRPC6 was investigated to explore the potential mechanism(s) of this process.

## 2 MATERIALS AND METHODS

### 2.1 Animal experimental design

Twelve‐week‐old diabetic male db/db mice were used for the animal experiments, and week‐matched db/m mice were used as a control. These mice were purchased from the Aier Matt Experimental Animal Company (Suzhou, China) and were randomly divided into four groups (n = 6 in each group): control (db/m), db/db, db/db plus 0.5 mg/kg TAC (a gift from Huadong Pharmaceutical Co., Ltd, Hangzhou, China) and db/db plus 1.0 mg/kg TAC. They were housed in Second Xiangya Hospital of Central South University using a 12‐hour/12‐hour light/dark cycle. All mice were allowed free access to tap water and standard laboratory chow (Second Xiangya Hospital of Central South University). Chow contains ≥18% protein, ≤10% water, ≥4% fat, ≤5% fibre, ≤8% ash, 1.0%‐1.8% calcium and 0.6%‐1.2% calcium. TAC was dissolved in 0.5% sodium carboxymethylcellulose (CMC‐Na, Sigma‐Aldrich, St. Louis, MO, USA) and was administered intragastrically to db/db mice. Untreated db/db and db/m mice received identical intragastric administration of the CMC‐Na for 8 weeks. Administration routes and drug doses were selected based on previous studies.^16^, ^17^, ^18^, ^22^, ^24^ Experimental mice were euthanized with an intraperitoneal injection of 50 mg/kg bodyweight sodium pentobarbital at 20 weeks, and samples of serum, urine and kidney tissues from each group were collected for further studies. The Animal Care and Use Committee of Second Xiangya Hospital of Central South University approved all animal procedures.

### 2.2 Assessment of biological chemistry parameters and urine albuminuria excretion

The blood glucose levels were detected twice a week with the blood sample withdrawn from mouse tail vein using the blood glucose monitor and test strips (Boehringer Mannheim, Mannheim, Germany). First, insert the blood glucose test strip into the blood glucose monitor. Then, fix the tail of the mouse and pierce the tail blood vessels of the mouse with a blood collection needle, and gently squeeze the centrifugal end with the hand. Last, the blood was transferred to test strips and the blood glucose value was recorded. Serum creatinine was assayed by standard automated enzymatic methods (Hitachi 912 automated analyzer, Hitachi, Mannheim, Germany).

Prior to killing, mice were placed in metabolic cages for collection of urine over 24‐hour period of time. After centrifugation (4°C, 3000 *g*, 10 minutes), the supernatant from urine sample was frozen at −70°C for subsequent analysis. Urine albumin concentration was analysed using urine albumin ELISA kit (Bethyl Laboratories, Montgomery, TX, USA). Urinary albumin excretion (ACR) was calculated as the urine albumin/creatinine ratio.

### 2.3 Morphological analysis and immunohistochemistry assay of kidneys in type 2 diabetic patients and experimental mice

Human kidney biopsy sections were obtained from DN patients (n = 10), and an equal number of patients with glomerular minor lesion (n = 10) were recruited for the study as controls as previously described.^25^ According to the pathological classification of DN,^26^ the DN patients observed in this study were class IIb (n = 4) and III (n = 6). All of the DN patients use metformin combined with insulin to control blood glucose. Patients who used adrenal cortical hormones or immunosuppressive agents were excluded. All procedures followed were in accordance with the World Medical Association Declaration of Helsinki, and all subjects provided written informed consent. The human protocol was approved by the Ethics Committee of Second Xiangya Hospital, Central South University.

Four‐micrometre‐thick kidney tissue sections from type 2 DN patients and db/db mice were stained with haematoxylin‐eosin (HE), periodic acid‐Schiff (PAS) and Masson's trichrome staining. Interstitial fibrosis and tubular atrophy (IFTA) was scored (0, no IFTA; 1, <25% IFTA; 2, 25%–50% IFTA; 3, >50% IFTA), and glomerular injury was analysed using a semiquantitative scoring system, as previously described.^25^, ^26^

Paraffin‐embedded kidney tissue sections were prepared for immunohistochemistry (IHC) analysis as previously described.^27^, ^28^ The sections were deparaffinized, rehydrated and placed in 0.1 mol/L sodium citrate (pH 6.0). Then, the sections were incubated with primary antibody against anti‐NFATc1 (Abcam, Cambridge, UK, ab175134), anti‐TRPC6 (Abcam, ab62461), anti‐fibronectin (Abcam, ab2413), anti‐collagen‐1 (Affinity, AF7001), anti‐IL‐6 (Proteintech, Wuhan, China, Cat.No.66146‐1‐Ig) or anti‐TNF‐α (Abcam, ab6671) overnight at 4°C and horseradish peroxidase‐conjugated secondary antibody for 1 hour at room temperature. Colour development was obtained using a diaminobenzidine (DAB) kit. There were more than 10 non‐overlapping images were taken for each kidney section at a magnification of ×400 (db/db mice) and ×200 (DN patients). Images were analysed using ImageJ. The threshold of images was adjusted to select the positive area (shown in red) and calculated the integral optical density (IOD) of each picture.

### 2.4 Analysis of renal apoptosis

Terminal deoxynucleotidyl transferase dUTP nick end‐labelling (TUNEL) staining was used to gauge apoptosis in the kidney sections of the mouse groups according to the manufacturer's instructions.^29^

### 2.5 Flow cytometry analysis of macrophage markers

Single renal cell suspensions obtained from db/db mice were blocked with antimouse CD45, CD11b and F4/80 in flow cytometry buffer (2% foetal bovine serum in phosphate buffer saline) for 30 minutes on ice in the dark, followed by flow cytometric analysis with FlowJo software (Tree Star Inc., Ashland, CA, USA). The data were analysed with FlowJo 10 software as previously described.^30^

### 2.6 Cell culture studies

The human proximal tubular epithelial cell line (HK‐2 cells) was purchased from ATCC (USA) and used for in vitro studies. Time‐dependent experiments were performed in HK‐2 cells using 30 mmol/L D‐glucose (high glucose, HG) for 0‐48 hours. HK‐2 cells were treated with various concentrations of TAC (50‐400 nmol/L) in HG conditions according to previous studies.^31^, ^32^, ^33^, ^34^ In addition, NFATc1 siRNA or a TRPC6 overexpression plasmid was transfected into the HK‐2 cells with or without TAC incubation, using Lipofectamine 2000 reagent (Life Technologies, USA) according to the manufacturer's instructions.^25^

### 2.7 Expression and translocation of NFATc1 and TRPC6 by immunofluorescence assay

HK‐2 cells received the above treatments and were then detected by an immunofluorescence (IF) assay. Briefly, HK‐2 cells were fixed, infiltrated, blocked and then incubated with anti‐NFATc1 or anti‐TRPC6 antibodies overnight at 4°C. The cell nuclei were stained with 4′, 6‐diamidino‐2‐phenylindole (DAPI), and images were obtained with confocal microscopy as described previously.^27^, ^28^

### 2.8 Real‐time polymerase chain reaction (PCR) analysis

Total RNA from renal sections and HK‐2 cells was isolated using TRIzol (Invitrogen, Carlsbad, CA, USA). First‐strand cDNA was synthesized using the Takara kit. Real‐time PCR was performed with SYBR GreenER qPCR SuperMix (Thermo Fisher Scientific, Waltham, MA, USA) on A Light Cycler 96 System (Roche, Roche, Switzerland). The mRNA levels of NFATc1 and TRPC6 were normalized to β‐actin. The primer sequences used for amplification are shown in Table 1.

**Table 1:** Primer sequences used for qRT‐PCR

Gene	Species	Primer sequence (5′‐3′)	Amplicon size (bp)
NFATc1	Mouse	Forward: GGTGCCTTTTGCGAGCAGTATC	142
		Reverse: CGTATGGACCAGAATGTGACGG	
TRPC6	Mouse	Forward: GCAGTGAAGTTCCTCGTGGT	151
		Reverse: GGAAAATGGTGAAGGAGGCTG	
β‐ACTIN	Mouse	Forward: GGCTGTATTCCCCTCCATCG	154
		Reverse: CCAGTTGGTAACAATGCCATGT	
NFATc1	Human	Forward: CACCAAAGTCCTGGAGATCCCA	132
		Reverse: TTCTTCCTCCCGATGTCCGTCT	
TRPC6	Human	Forward: GCAGACAATGGCGGTCAAGTTC	157
		Reverse: AATGGTGAAGGAGGCTGCGTGT	
β‐ACTIN	Human	Forward: CATGTACGTTGCTATCCAGGC	88
		Reverse: CTCCTTAATGTCACGCACGAT	

Abbreviations: NFATc1, nuclear factor of activated T cell c1; TRPC6, transient receptor potential channel C6.

### 2.9 Protein extraction and Western blot analysis

Protein extraction from mouse kidney tissues and HK‐2 cells was performed with radio immunoprecipitation assay (RIPA) lysis buffer. Nucleoprotein and plasma protein extraction was performed using the nuclear and cytoplasmic protein extraction kits (Beyotime Institute of Biotechnology, China) according to the manufacturer's instructions. The protein concentration was determined by using a BCA protein assay kit (Thermo Fisher Scientific). Proteins were separated using 10% SDS‐PAGE and transferred to PVDF membranes, which were probed with primary antibodies against NFATc1 (1:1000 dilution, Abcam), TRPC6 (1:1000 dilution, Abcam), fibronectin (1:1000 dilution, Abcam), collagen 1 (1:1000, Affinity), cleaved caspase 3 (1:1000, CST) and IL‐6 (1:1000 dilution, Proteintech). β‐actin (1:5000, Proteintech) and lamin B1 (1:5000, Proteintech) were used as internal controls. Antibody‐antigen complexes were detected with enhanced chemiluminescence (ECL) (Amersham Pharmacia Biotech, Uppsala, Sweden) after incubation with a suitable secondary antibody as described previously.^27^, ^28^

### 2.10 Statistical analysis

All statistical analyses were performed using SPSS 20.0 software. Values are presented as the mean ± SD and were assessed by Student's *t* test or one‐way analysis of variance. Correlation analysis was performed using Pearson's correlation analysis. *P* < .05 indicated a significant difference.

## 3 RESUTLS

### 3.1 Protective effect of TAC on renal function and morphological changes in the kidneys of db/db mice

**Figure 1:**
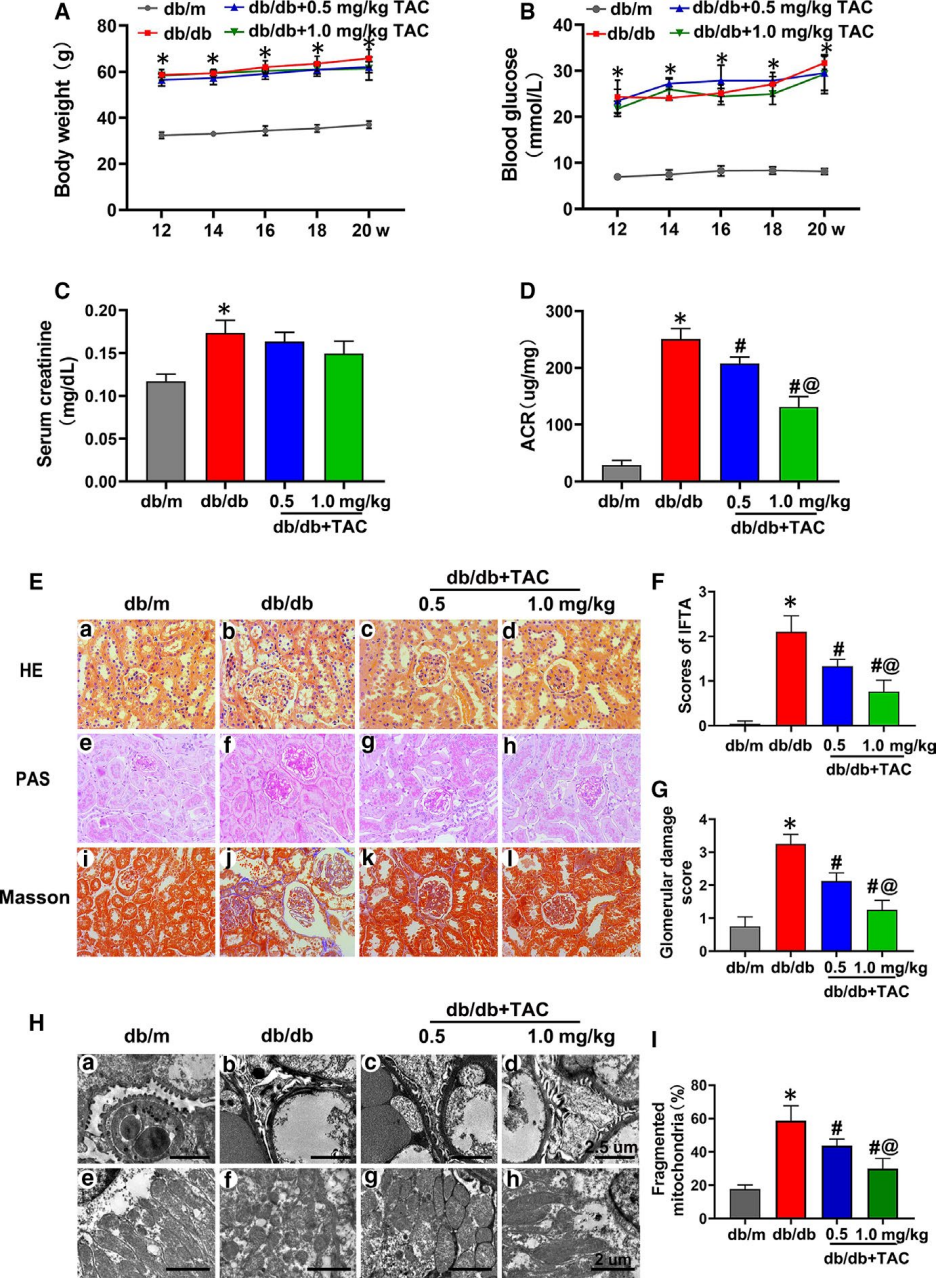
Tacrolimus (TAC) ameliorated tubulointerstitial injury in db/db mice. A, Bodyweight changes in db/db mice with or without TAC (0.5 or 1.0 mg/kg) for 12‐20 wk. B, Blood glucose concentrations in each group. C, Serum creatinine level. D, Urinary albumin excretion (ACR). E, Haematoxylin‐eosin **(**HE) (a‐d), periodic acid‐Schiff (PAS) (e‐h) and Masson's staining (i‐l) of renal tissues from the mice. F, G, Quantitative analysis of interstitial fibrosis and tubular atrophy (IFTA) scores and glomerular damage in the kidneys in each group. H, I, Transmission electron microscopy (TEM) showing thickening of the basement membrane and mitochondrial fragmentation in the kidneys of db/db mice compared with those of db/m mice, which were ameliorated by TAC in a dose‐dependent manner. The values are the mean ± SD, *P < .05 vs db/m;^ #^ *P* < .05 vs db/db; ^@^ *P* < .05 vs db/db + 0.5 mg/kg TAC mice. n = 6

The bodyweight and serum creatinine levels notably increased in db/db mice compared with those of db/m mice, while no significant difference was observed with TAC treatment (Figure 1A,C). The blood glucose level was slightly higher in 0.5 mg/kg TAC treatment group as compared to db/db mice. There was no aggravated effect on blood glucose level of db/db mice as the dose of TAC treated from 0.5 to 1.0 mg/kg (Figure 1B). The level of ACR was significantly increased in db/db mice, whereas it was down‐regulated by TAC treatment, especially in high dose TAC treatment group (Figure 1D). In addition, HE, PAS and Masson's staining showed that morphological changes in the kidney were also attenuated with TAC treatment, which was characterized by amelioration of glomerular hypertrophy, mesangial matrix proliferation, tubular atrophy and tubulointerstitial fibrosis (Figure 1E). Subsequent semiquantification analysis and glomerular and tubulointerstitial scores verified these results (Figure 1F,G). Transmission electron microscopy (TEM) showed basement membrane thickening, podocyte foot process fusion and mitochondrial fragmentation in tubular cells. Encouragingly, TAC alleviated these pathological changes in a dose‐dependent manner (Figure 1H,I).

### 3.2 Tubulointerstitial injury in db/db mice was attenuated by TAC

**Figure 2:**
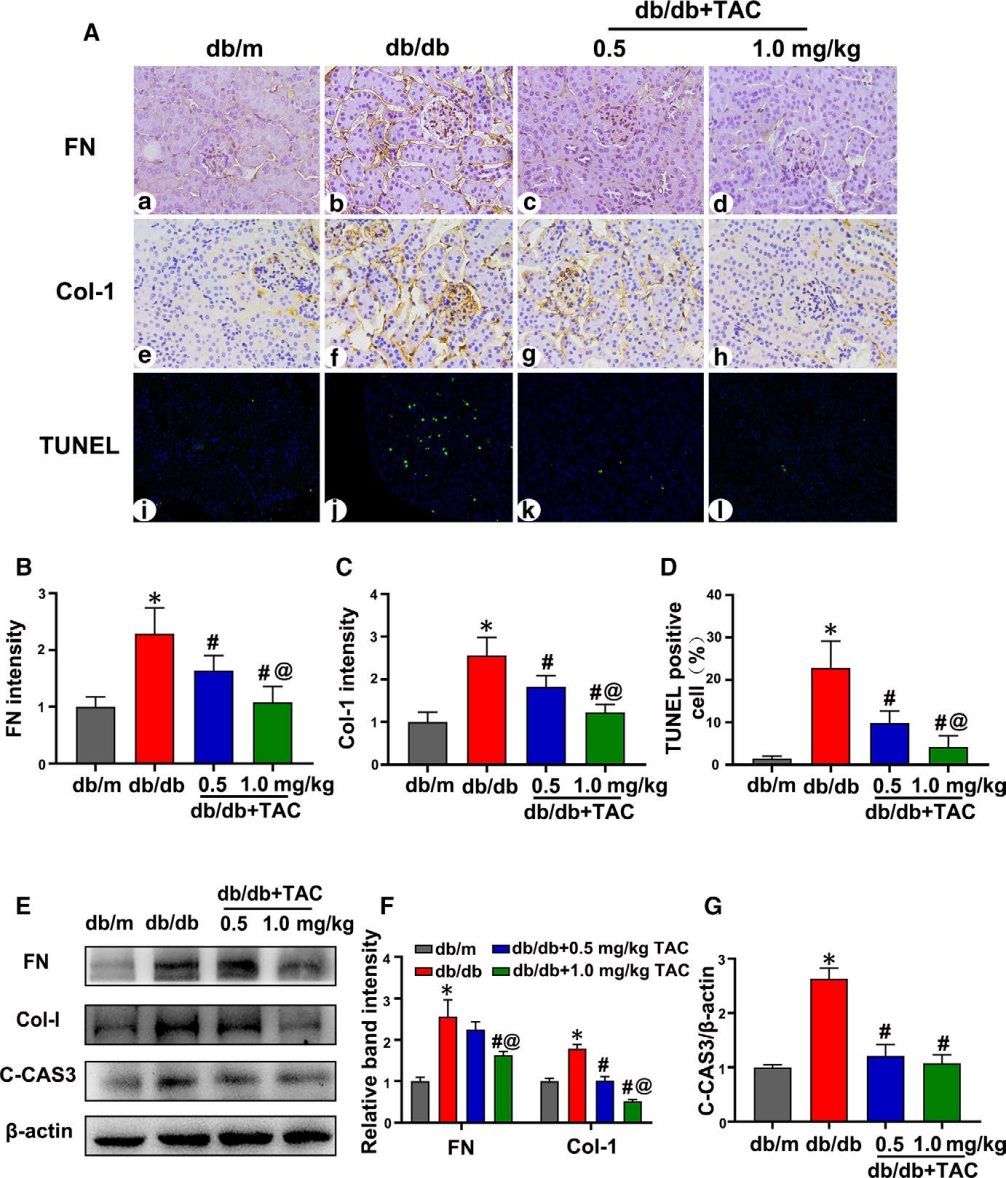
Tacrolimus (TAC) reduced tubulointerstitial fibrosis and apoptosis in db/db mice. A, Fibronectin (FN) (a‐d) and collagen 1 (Col‐1) (e‐h) expression levels were assessed by IHC (magnification × 400). Apoptosis in kidney tubular cells was detected with TUNEL staining (i‐l). B, C, Semiquantification of FN and Col‐1 intensity. n = 6 per group. D, Statistical analysis of TUNEL‐positive cells. n = 6 per group. E, Western blot analysis of FN, Col‐1 and cleaved caspase 3 (C‐CAS3) expression; β‐actin was used as a control. F, G, Relative band density of FN, Col‐1 and C‐CAS3. n = 3 per group. The values are the mean ± SD, *P < .05 vs db/m; ^#^ *P* < .05 vs db/db; ^@^ *P* < .05 vs db/db + 0.5 mg/kg TAC mice. IHC, immunohistochemistry; TUNEL, terminal deoxynucleotidyl transferase dUTP nick end‐labelling

Immunohistochemistry (IHC) showed that fibronectin (FN) and collagen 1 (Col‐1) intensity in the kidney tissues of db/db mice was attenuated by TAC in a dose‐dependent manner (Figure 2Aa‐h, B and C), and these findings were confirmed by Western blotting (Figure 2E,F). In addition, TUNEL‐positive cells in the kidneys of db/db mice were decreased with the treatment of 0.5 mg/kg TAC and were augmented in the 1.0 mg/kg TAC group (Figure 2A i‐l and D). Similarly, TAC also reduced the expression of cleaved caspase 3 (C‐CAS3) in the kidneys of db/db mice (Figure 2E), which was verified by densitometry analysis (Figure 2G).

### 3.3 Macrophage infiltration and the level of inflammatory cytokines in db/db mice were ameliorated by TAC

**Figure 3:**
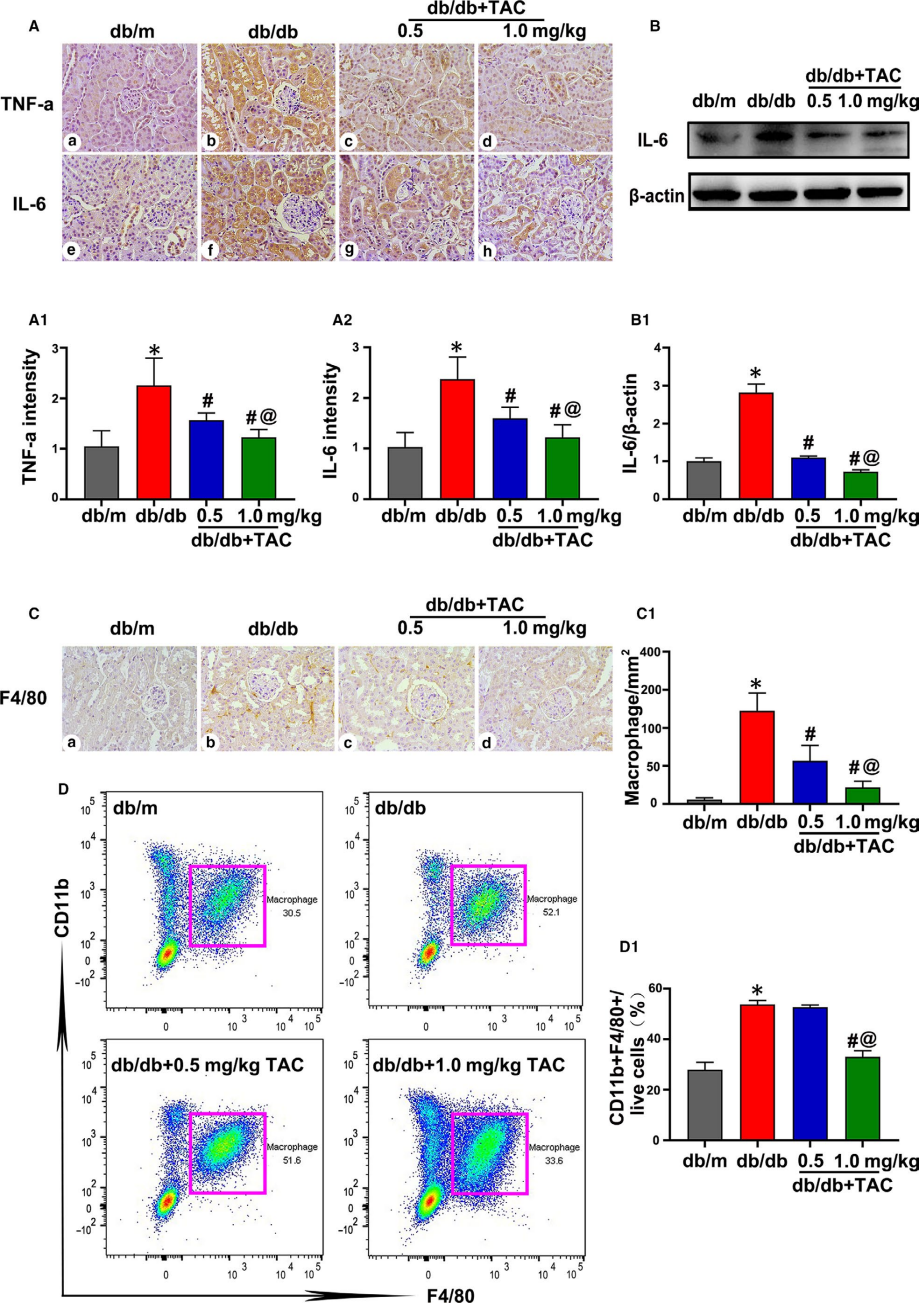
Tacrolimus (TAC) inhibited macrophage infiltration and pro‐inflammatory cytokine protein expression in the kidneys of db/db mice. A, Representative immunohistochemistry (IHC) images of TNF‐α (a‐d) and IL‐6 (e‐h) in the mouse kidney sections (magnification × 400). A1, A2, Semiquantification of IHC staining of TNF‐α and IL‐6. n = 6 per group. B, Western blot analysis of IL‐6 expression in the kidney tissue. The expression of β‐actin was used as a control. n = 3 per group. B1, Relative band density of IL‐6. C, Representative IHC staining of F4/80 to show macrophages. n = 6 per group. C1, Quantitative analysis of macrophages. D, D1, Flow cytometric analysis of CD11b^+^F4/80^+^ macrophages in the renal tissues from the different groups. n = 3 per group. The values are the mean ± SD, *P < .05 vs db/m; ^#^ *P* < .05 vs db/db; ^@^ *P* < .05 vs db/db + 0.5 mg/kg TAC mice

In addition to the effect of TAC on tubulointerstitial injury, changes in inflammation after treatment were also investigated. As shown in Figure 3A, the intensity of IL‐6 and TNF‐α expression was significantly up‐regulated in the kidney tissues of db/db mice compared to that of db/m mice. Treatment with TAC dramatically reduced the intensity of IL‐6 and TNF‐α expression (Figure 3A, A1 and A2). A similar effect on IL‐6 protein expression was shown by Western blotting (Figure 3B,B1).

Macrophages are crucial for kidney inflammation in DN. Thus, macrophage infiltration was assessed by IHC analysis of F4/80 and flow cytometry assays. It was shown that the intensity of F4/80 obviously increased in the kidney tissues of db/db as compared to that of db/m mice, while this effect was dramatically inhibited by the TAC treatment (Figure 3C,C1). Flow cytometry revealed an obvious increase in the number of CD11b^+^F4/80^+^ macrophages in db/db mice compared with that of db/m mice. However, a high dose of TAC significantly decreased the number of CD11b^+^F4/80^+^ macrophages (Figure 3D,D1). These data indicate that TAC has a beneficial effect on tubulointerstitial inflammation under DN conditions.

### 3.4 Overexpression of NFATc1 and TRPC6 in the kidney tissues of db/db mice was inhibited by TAC treatment

**Figure 4:**
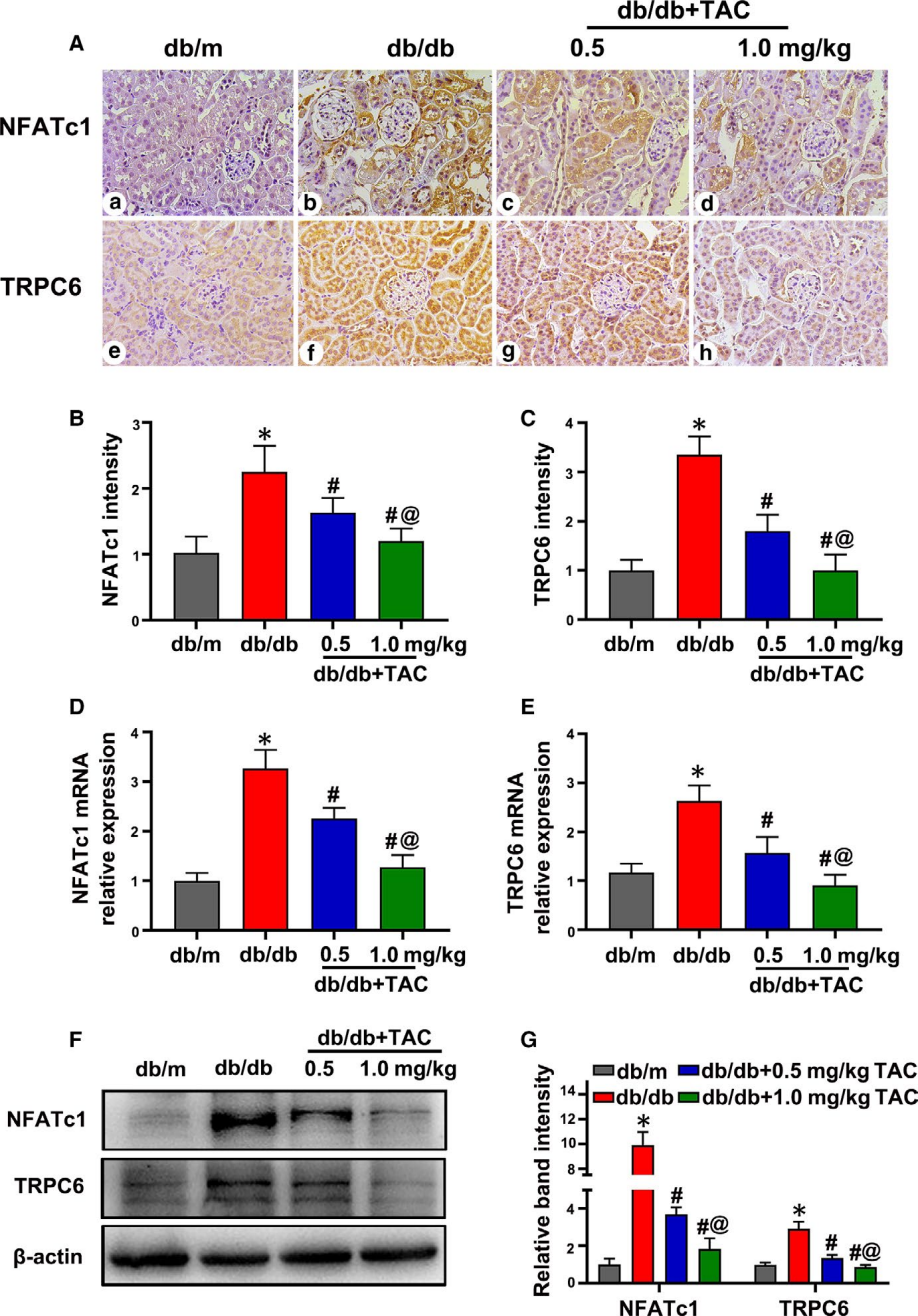
Up‐regulated expression of nuclear factor of activated T cell 1 (NFATc1) and transient receptor potential channel 6 (TRPC6) in the kidney sections of db/db mice was attenuated with tacrolimus (TAC) treatment. A, Representative IHC images of NFATc1 and TRPC6 in the kidneys of mice. B, C, Semiquantification analysis of NFATc1 and TRPC6 expression determined by IHC. n = 6 per group. D, E, Real‐time PCR showed the relative mRNA expression of NFATc1 and TRPC6. n = 6 per group. F, Western blot analysis of NFATc1 and TRPC6. n = 3 per group. G, Relative band density of NFATc1 and TRPC6. The values are normalized by β‐actin. Values are the mean ± SD, *P < .05 vs db/m; ^#^ *P* < .05 vs db/db; ^@^ *P* < .05 vs db/db + 0.5 mg/kg TAC mice. Real‐time PCR, real‐time polymerase chain reaction; IHC, immunohistochemistry

Immunostaining showed that the intensity of NFATc1 and TRPC6 was low in the kidneys of db/m mice, whereas expression was obviously increased in db/db mice, especially in the renal proximal tubules. With the administration of TAC, the high expression of NFATc1 and TRPC6 in the kidneys of db/db mice was partially normalized (Figure 4A‐C). Real‐time PCR showed a significant up‐regulation of NFATc1 and TRPC6 mRNA expression in the kidneys of db/db mice, while this up‐regulation was reversed by treatment with TAC (Figure 4D,E). A similar result was shown by Western blotting (Figure 4F,G).

### 3.5 NFATc1 and TRPC6 expression in kidney sections positively correlated with inflammation and tubular injury in type 2 DN patients

**Figure 5:**
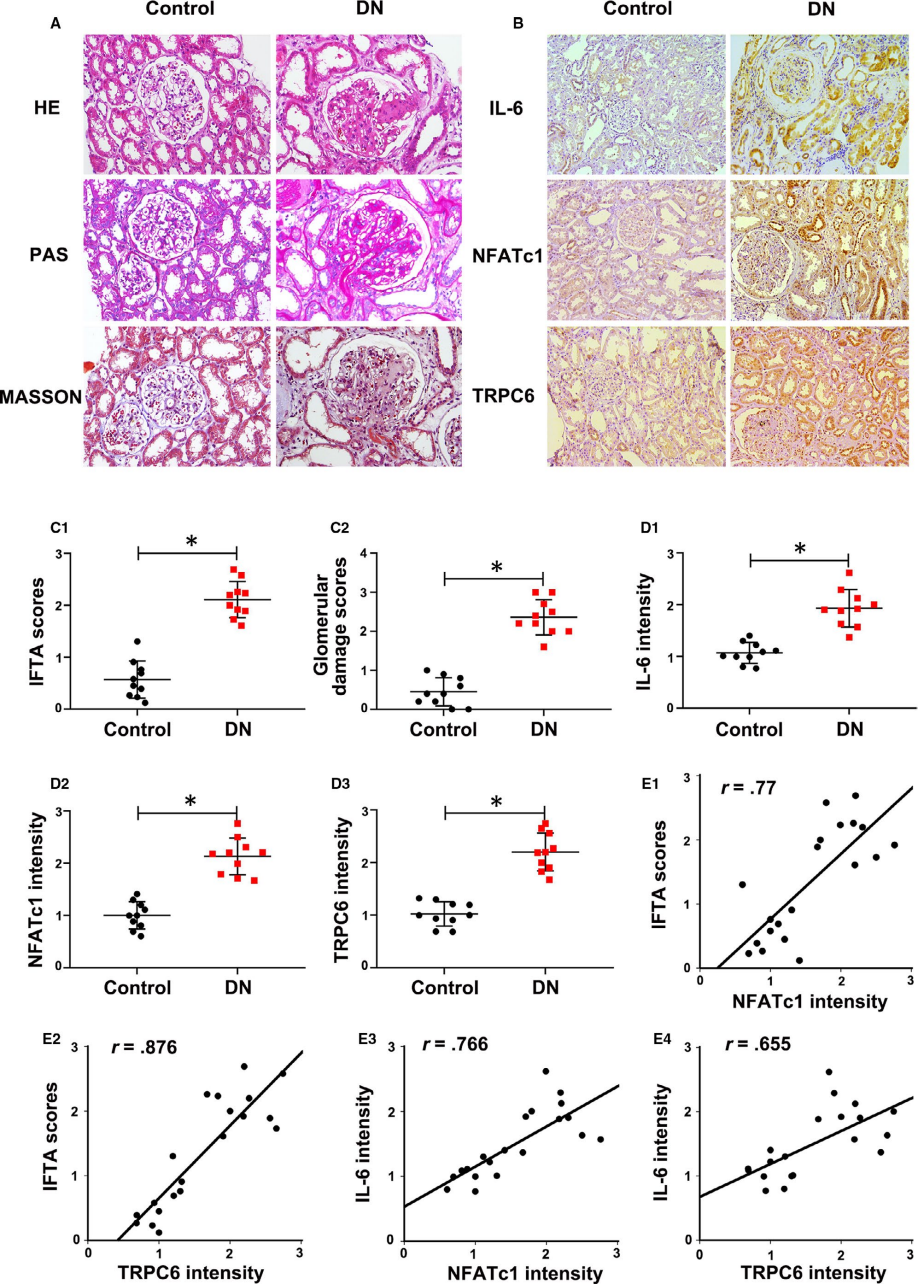
Increased nuclear factor of activated T cell 1 (NFATc1) and transient receptor potential channel 6 (TRPC6) expression positively correlated with tubulointerstitial inflammation in the kidney biopsy sections of type 2 diabetic nephropathy (DN) patients. A, Histological changes as shown by Haematoxylin‐eosin (HE), periodic acid‐Schiff (PAS) and Masson's staining. B, Immunohistochemistry (IHC) staining of IL‐6, NFATc1 and TRPC6. C1, C2, Quantitative analysis of interstitial fibrosis and tubular atrophy (IFTA) scores and glomerular injury. D1‐D3, Relative intensity of IL‐6, NFATc1 and TRPC6 in the kidney tissues of type 2 DN patients. E1‐E2, The correlations of NFATc1 and TRPC6 expression levels with IFTA scores. E3‐E4, The correlations between NFATc1, TRPC6 and IL‐6 expression in the kidney of type 2 DN patients. The values are the mean ± SD. *P < .05 vs control group. *r*: correlation coefficient. n = 10

The basal clinical data of the DN patients and controls in this study were shown in Table 2. HE, PAS and Masson's staining of the renal sections of DN patients showed mesangial matrix proliferation, tubular atrophy and interstitial fibrosis (Figure 5A). In addition, the IFTA and glomerular damage scores of patients with DN were higher than those of control subjects (Figure 5C1,C2).

**Table 2:** Clinical characteristics

	Control (n = 10)	DN (n = 10)
Age (y)	38.0 ± 12.4	50.2 ± 8.3
Sex (male/female)	6/4	5/5
Duration (y)	‐	11.9 ± 3.7
BMI (kg/m^2^)	23.1 ± 2.5	24.2 ± 3.2
Glucose (mmol/L)	4.6 ± 0.5	8.3 ± 4.1*
Hb (g/L)	130.2 ± 13.6	128.8 ± 11.5
HbA_1C_ (%)	4.5 ± 0.4	7.6 ± 0.5*
Total cholesterol (mmol/L)	4.9 ± 0.8	5.8 ± 0.6
Triglyceride (mmol/L)	1.7 ± 0.3	2.3 ± 0.4*
Albumin (g/L)	37.3 ± 3.6	26.4 ± 2.3*
SCr (μmol/L)	62.5 ± 8.9	127.2 ± 18.7*
BUN (mmol/L)	5.2 ± 0.7	8.5 ± 1.9*
UA (μmol/L)	295.5 ± 21.2	372 ± 36.6*
24‐h urine protein (g/d)	0.8 ± 0.5	4.9 ± 1.4*
SBP (mm Hg)	112 ± 5.6	116 ± 7.4
DBP (mm Hg)	72 ± 7.4	80 ± 10.5

Abbreviations: BMI, body mass index; BUN, blood urea nitrogen; DBP, diastolic blood pressure; Hb, haemoglobin; HbA_1C_, glycosylated haemoglobin; SBP, systolic blood pressure; SCr, serum creatinine; UA, uric acid.

* *P* < .05 vs control; values are Mean ± SD.

IHC staining revealed increased expression of NFATc1 and TRPC6 as well as IL‐6 in DN patients compared with that of controls (Figure 5B, D1‐D3). In addition, it was shown that the intensity of NFATc1 and TRPC6 expression was positively correlated with the IFTA scores (*r* = 0.77, *r* = 0.876, *P* < .05) (Figure 5E1,E2). Moreover, the expression of NFATc1 and TRPC6 were also positively correlated with IL‐6 expression (*r* = 0.766, *r* = 0.655, *P* < .05) (Figure 5E3,E4), suggesting that high levels of NFATc1 and TRPC6 expression are tightly correlated with tubular injury and inflammation of kidney in type 2 DN patients.

### 3.6 TAC alleviated inflammation and apoptosis in HK‐2 cells under hyperglycaemia conditions

**Figure 6:**
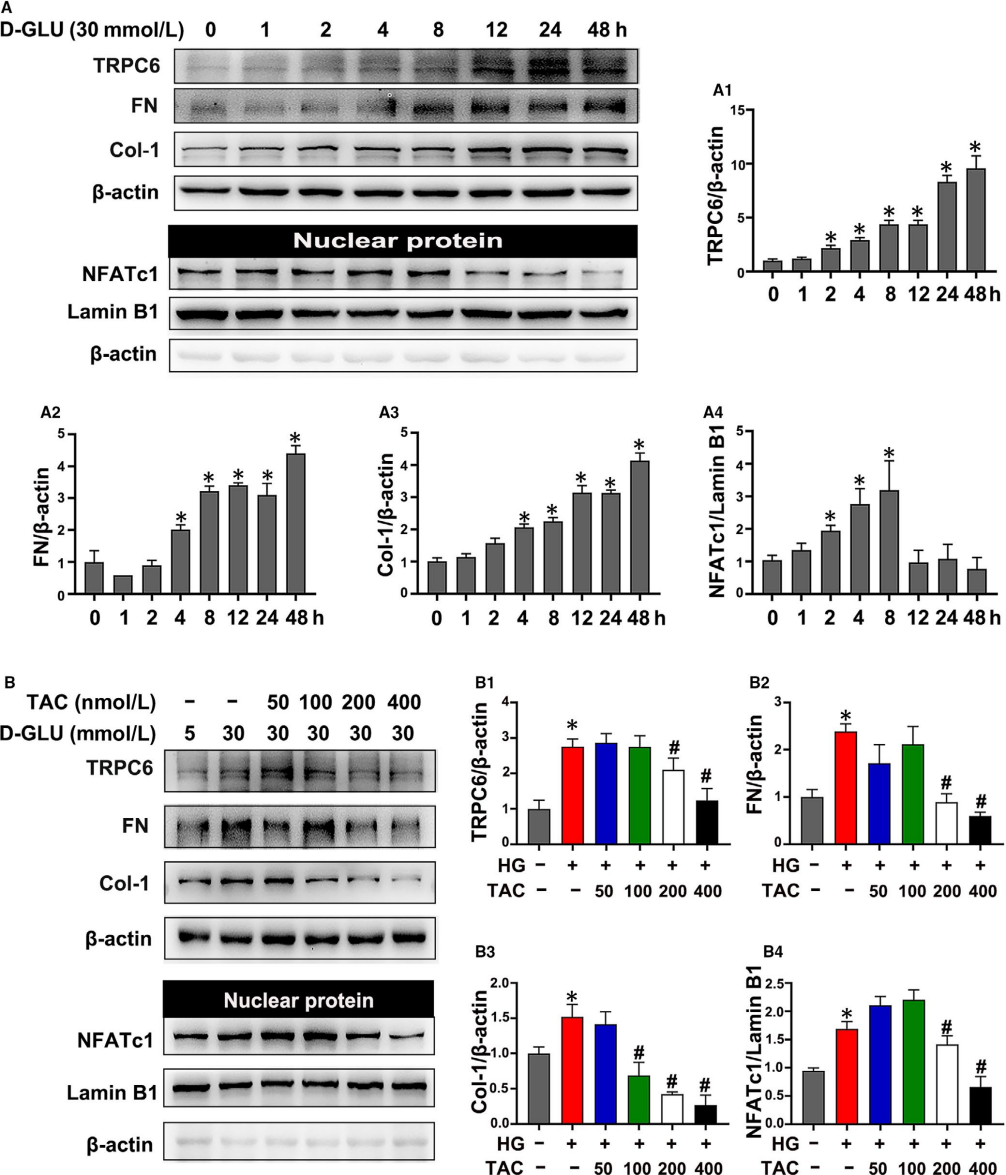
Tacrolimus (TAC) suppressed nuclear factor of activated T cell 1 (NFATc1), transient receptor potential channel 6 (TRPC6) and extracellular matrix protein in HK‐2 cells incubated with high glucose (HG). A, Western blot analysis of TRPC6, fibronectin (FN), collagen 1 (Col‐1) and nuclear NFATc1 expression in HK‐2 cells with 30 mmol/L HG exposure for 0‐48 h. A1‐A4, Quantification of the average Western blot band intensity. B, The expression of TRPC6, FN, Col‐1 and nuclear NFATc1 in HK‐2 cells incubated with 30 mmol/L HG and 50‐400 nmol/L TAC was detected by Western blot. B1‐B4, Quantification of the average Western blot band intensity. The values are presented as the mean ± SD, *P < .05 vs LG; ^#^ *P* < .05 vs HG. n = 3

**Figure 7:**
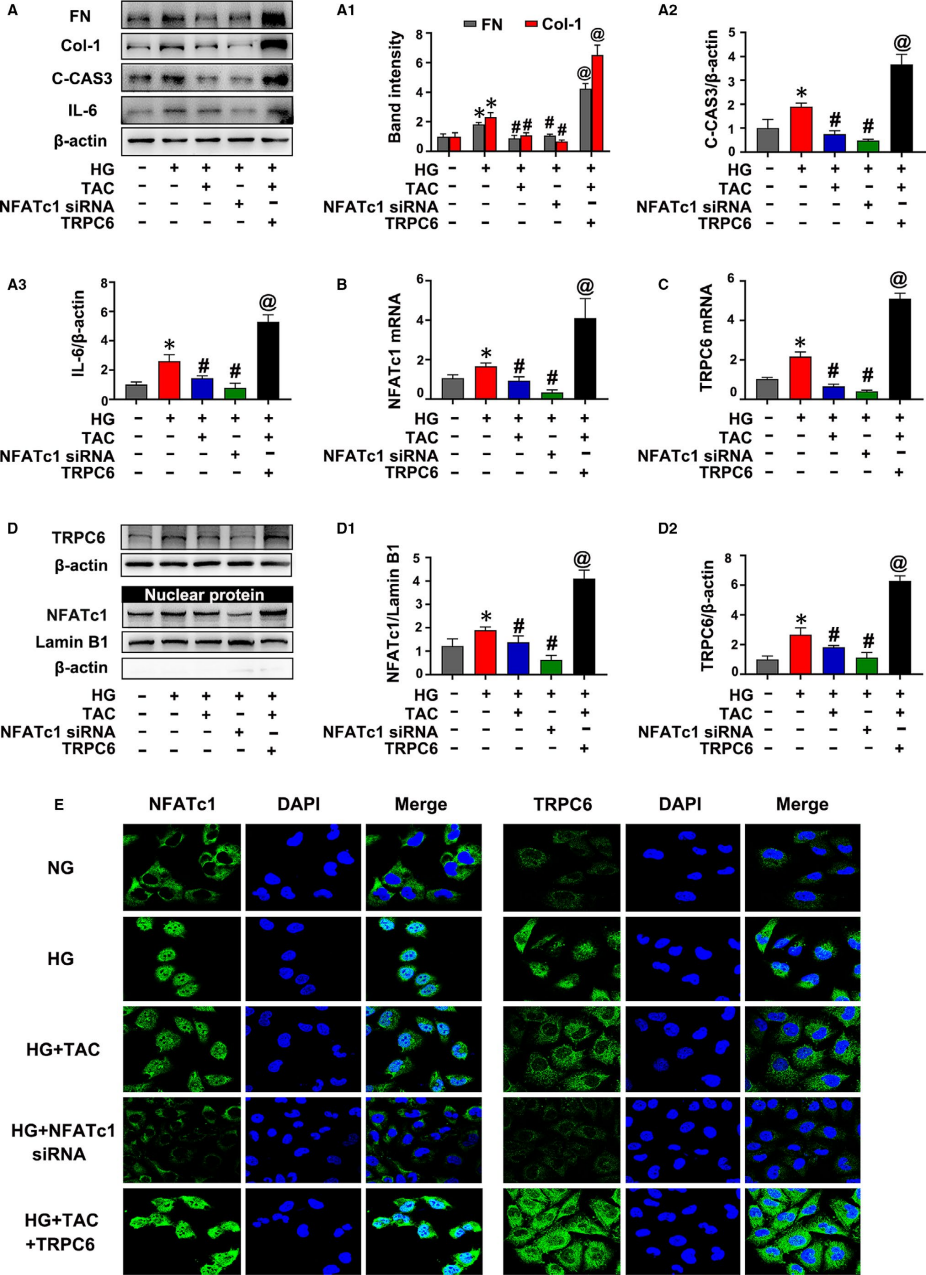
NFATc1 (nuclear factor of activated T cell 1)/TRPC6 (transient receptor potential channel 6) contributed to high glucose (HG)‐induced inflammation and apoptosis in HK‐2 cells treated with tacrolimus (TAC). A, The expression of IL‐6, fibronectin (FN), collagen 1 (Col‐1) and cleaved caspase 3 (C‐CAS3) in HK‐2 cells incubated with TAC or NFATc1 siRNA and treated with or without the TRPC6 overexpression plasmid under HG conditions was detected by Western blot. A1‐A3, Quantification of the average Western blot band intensity. B, C, The expression of NFATc1 and TRPC6 by real‐time PCR. D, Western blot analysis of TRPC6 and nuclear NFATc1 expression. D1‐D2, Quantification of the average intensity of NFATc1 and TRPC6 by Western blotting. E, Immunofluorescence staining of NFATc1 and TRPC6. The values are presented as the mean ± SD, *P < .05 vs low‐glucose (LG); ^#^ *P* < .05 vs HG; ^@^ *P* < .05 vs HG + TAC. n = 3. Real‐time PCR, real‐time polymerase chain reaction

As shown in Figure 6A, Western blotting showed a significant time‐dependent increased in FN and Col‐1 in HK‐2 cells incubated with 30 mmol/L HG (Figure 6A, A2 and A3). After treatment with various doses of TAC (50‐400 nmol/L), the HG‐induced high expression of FN and Col‐1 was significantly inhibited in HK‐2 cells in a dose‐dependent manner (Figure 6B, B2 and B3). Similarly, Western blotting showed up‐regulated IL‐6 and C‐CAS3 in HK‐2 cells exposed to HG, which was reversed by TAC treatment (Figure 7A, A2 and A3), indicating that TAC exerts a beneficial role in inflammation, production of extracellular matrix and apoptosi**s** of HK‐2 cells under hyperglycaemia.

### 3.7 TAC inhibited HG‐induced high expression of NFATc1 and TRPC6 in HK‐2 cells

It was shown that an increased expression of TRPC6 in HK‐2 cells was induced by HG in a time‐dependent manner (Figure 6A,A1). Western blotting of nuclear protein extracts revealed a significant increase in nuclear NFATc1 expression in HK‐2 cells incubated with HG, which reached a peak at 8 hours, and then decreased at 12 hours (Figure 6A,A4). In addition, the HG‐induced up‐regulated expression of TRPC6 and nuclear NFATc1 was significantly inhibited by treatment with 200‐400 nmol/L TAC, while there was no obvious effect in the groups that received a low dose of TAC (50‐100 nmol/L) (Figure 6B, B1 and B4). As a result, 400 nmol/L TAC was used for further experiments.

### 3.8 NFATc1/TRPC6 mediated the effect of TAC on inflammation and apoptosis of HK‐2 cells under HG conditions

As shown in Figure 7A, TAC inhibited the high expression of FN, Col‐1 and C‐CAS3 in HK‐2 cells incubated with HG. Similarly, transfection of NFATc1 siRNA also inhibited HG‐induced expression of FN, Col‐1 and C‐CAS3 in HK‐2 cells. However, the effect of TAC was abolished with the TRPC6 overexpression plasmid (Figure 7A, A1 and A2). In addition, TAC or transfection of NFATc1 siRNA also down‐regulated IL‐6 protein expression in HK‐2 cells incubated with HG, whereas the TRPC6 overexpression plasmid blocked the inhibitory effect of TAC on IL‐6 protein expression (Figure 7A,A3).

It was observed that HG‐induced increased NFATc1 mRNA and protein expression in HK‐2 cells, but this effect was inhibited by TAC or transfection of NFATc1 siRNA (Figure 7B, D and D1). However, transfection of the TRPC6 overexpression plasmid dramatically blocked the inhibitory effect of TAC on NFATc1 expression (Figure 7B, D and D1). On the other hand, TAC or NFATc1 siRNA decreased the expression of TRPC6 (Figure 7C, D and D2). IF analysis showed that the intensity of TRPC6 increased in HK‐2 cells with HG incubation, while it was partially blocked by TAC or NFATc1 siRNA transfection (Figure 7E). TAC or NFATc1 siRNA transfection partially blocked the intensity and nuclear translocation of NFATc1 in HK‐2 cells induced by HG. However, transfection of the TRPC6 plasmid abolished the inhibitory effect of TAC and exacerbated the high expression and translocation of NFATc1 (Figure 7E). These results indicate that NFATc1/TRPC6 mediates the effect of TAC on inflammation and there is a positive feedback loop between NFATc1 and TRPC6 in HK‐2 cells under HG conditions.

## 4 DISCUSSION

The present study delineated that administration of TAC exerted a beneficial role in tubulointerstitial injury, especially in inflammation of db/db mice, which was characterized by suppressed tubular atrophy and tubulointerstitial fibrosis, reduced macrophage infiltration and inflammatory cytokine (IL‐6, TNF‐a) expression. Moreover, in vitro experiment also demonstrated that TAC ameliorated inflammation, the production of extracellular matrix and apoptosis of HK‐2 cells exposed to exogenous HG. Further experiments suggested that NFATc1 siRNA mimicked the effect of TAC, whereas transfection of the TRPC6 overexpression plasmid blocked the effect of TAC on tubular inflammation under hyperglycaemia. Taken together, these data indicate a potential protective effect of TAC on tubulointerstitial inflammation and injury, which is mediated by the NFATc1/TRPC6 pathway.

It has been demonstrated that inflammation is crucial for the pathogenesis and progression of DN.^6^, ^35^, ^36^ Renal pathological analysis revealed that macrophages are the most abundant immune cell in DN patients and animal models, which correlates with tubular atrophy and tubulointerstitial fibrosis.^37^, ^38^, ^39^ Macrophage depletion in diabetic mice ameliorated albuminuria and morphological changes in DN.^40^ Notably, accumulating data suggested that tubular cells act as a driving force rather than as ‘victims’ in the inflammation in DN. It was found that MCP‐1 increased in HK‐2 cells exposed to HG, which enhanced the migration ability of co‐cultured macrophage. However, inhibition of miR‐37α decreased the expression of MCP‐1 in HK‐2 cells under HG condition, which inhibited the migration of macrophage and the secretion of IL‐6 and IL‐18.^11^ In addition, HG could increase macrophage inflammatory protein expression in proximal tubular cells via KCa3.1 channel activation.^41^ However, inhibition of sodium‐glucose cotransporter 2 (SGLT2), which was located on the early segments of the proximal tubule, significantly decreased renal inflammation and lipid accumulation in db/db mice.^42^ Moreover, we previously observed that the exchange protein directly activated by cAMP (Epac) agonist reduced the expression of MCP‐1 in HK‐2 cells, which abolished macrophage migration using a double chamber assay (W. X. Yang, et al. unpublished data), indicating that tubular cells play a critical role in the inflammatory cascade under HG conditions.

TAC, a calcineurin inhibitor, could reduce the phosphorylation activity of calcineurin by binding to FK506 binding protein (FKBP12),^43^ and preventing the dephosphorylation and nuclear translocation of NFAT,^12^ and has been widely used in immune‐associated kidney diseases.^14^, ^44^, ^45^ Emerging data demonstrated that TAC exerts a beneficial effect on proteinuria and fibrosis,^15^, ^16^ especially against podocyte injury in DN.^17^, ^22^ It was found that TAC reduces calpain‐mediated NFAT activation, which reduces tubular cell death and the transcription of pro‐inflammatory cytokine genes in ischaemia‐reperfusion injury (IRI) mice.^46^ However, few studies have reported the effect of TAC on tubulointerstitial inflammation in DN.

In the present study, it was shown that TAC not only reduced proteinuria, and improved tubulointerstitial pathological changes, but also markedly ameliorated macrophage infiltration and pro‐inflammatory cytokine expression in db/db mice (Figures 1 and 3). Moreover, we observed that TAC has no obvious influence on blood glucose level of db/db mice, although a little higher in 0.5mg/kg TAC treatment (Figure 1). The result was in accordance with previous literatures in STZ‐induced diabetic rats.^16^, ^17^, ^18^, ^22^ Thus, we suppose that there was no severe glucotoxicity with the treatment of TAC under DN condition and TAC‐induced toxicity in pancreatic β‐cells is not a confounding factor of this experiment. Further study, especially randomized controlled trial, needs to verify in the future. In vitro experiments reduced inflammatory cytokine expression, extracellular matrix production and apoptosis in response to TAC treatment in HK‐2 cells incubated with exogenous HG (Figures 6 and 7). These finding indicate that TAC successfully inhibited tubular cell‐associated inflammation in DN, which provides important complementary data to previous studies on the protective effect of podocyte injury under DN conditions.^17^, ^22^

Next, we investigated whether NFAT inactivation contributed to the alleviation of tubulointerstitial inflammation in DN. It was shown that NFATc1 expression was significantly increased in the kidneys of both type 2 DN patients and db/db mice, which correlated with tubular injury and IL‐6 expression (Figures 4 and 5). In addition, NFATc1 expression and nuclear translocation were up‐regulated in HK‐2 cells incubated with HG, while these effects were dramatically reversed with TAC treatment (Figures 6 and 7). Notably, transfection of NFATc1 siRNA dramatically inhibited HG‐induced expression of IL‐6 in addition to FN, Col‐1 and cleaved caspase 3 in HK‐2 cells (Figure 7). These data suggest that NFATc1 might contribute to the effect of TAC on tubulointerstitial inflammation in DN.

Recently, emerging evidence has suggested that TRPC6 contributes to inflammation in tubular cells under DN conditions.^47^, ^48^ Fu YQ et al found that increased TRPC6 expression is associated with the release of IL‐8 and IL‐6 in HK‐2 cells incubated with HG.^47^ In addition, TRPC6 siRNA inhibited inflammation and proliferation of HK‐2 cells,^47^ and knockout of TRPC6 reduced proteinuria and tubular injury in Akita mice.^21^ In this study, TRPC6 expression was obviously up‐regulated in the kidneys of DN patients, which positively correlated with tubulointerstitial damage score and IL‐6 expression (Figure 5). In addition, TRPC6 expression increased in the kidney tissue of db/db mice and in HK‐2 cells incubated with HG, and these effects were reversed by TAC treatment (Figures 4, 6 and 7). However, the beneficial effect of TAC on tubular inflammation and apoptosis was partially neutralized by the TRPC6 overexpression plasmid (Figure 7), indicating TRPC6 contributes to the effect of TAC on the inflammation of tubular cells in DN.

Interestingly, it was shown that there was a tight correlation between NFAT and TRPC6.

TRPC6 is regulated by NFAT in cardiomyocytes and vice versa.^49^ Moreover, TRPC6 expression increased through positive feedback with NFAT in podocytes incubated with Ang II.^23^ Precisely, the promoter of the TRPC6 gene contains 2 conserved NFAT sites; therefore, upon NFAT binds to these domains, it could initiate the transcription of TRPC6.^49^ On the other hand, TRPC6, in turn, promotes the nuclear translocation of NFAT via calcium‐dependent activation of calcineurin, which enhances the dephosphorylation of NFAT.^23^, ^50^ In the present study, we found that HG‐induced TRPC6 expression was inhibited by NFATc1 siRNA or administration of TAC in HK‐2 cells. In addition, overexpression of TRPC6 in turn resulted in the nuclear translocation of NFAT (Figure 7D, D1, D2 and E), suggesting this feedback loop between NFATc1 and TRPC6 exists in tubular cells in DN, like in podocytes, cardiomyocytes and clear cell renal cell carcinoma (ccRCC) cells.^23^, ^49^, ^50^, ^51^

In addition to the extensive efforts, there are still many issues remain unclear. For example, there is no ideal animal mode to represent severe tubulointerstitial fibrosis, which was observed in some DN patients. So it still needs further clinical trial to evaluate the effect of TAC in DN patients in the future. Additionally, Jin et al found that TAC increased SGLT‐2 expression in kidney tissues of Sprague Dawley rats.^52^ Thus, it is an interesting topic and needed to confirm the relationship between TAC and SGLT2 in the future study.

**Figure 8:**
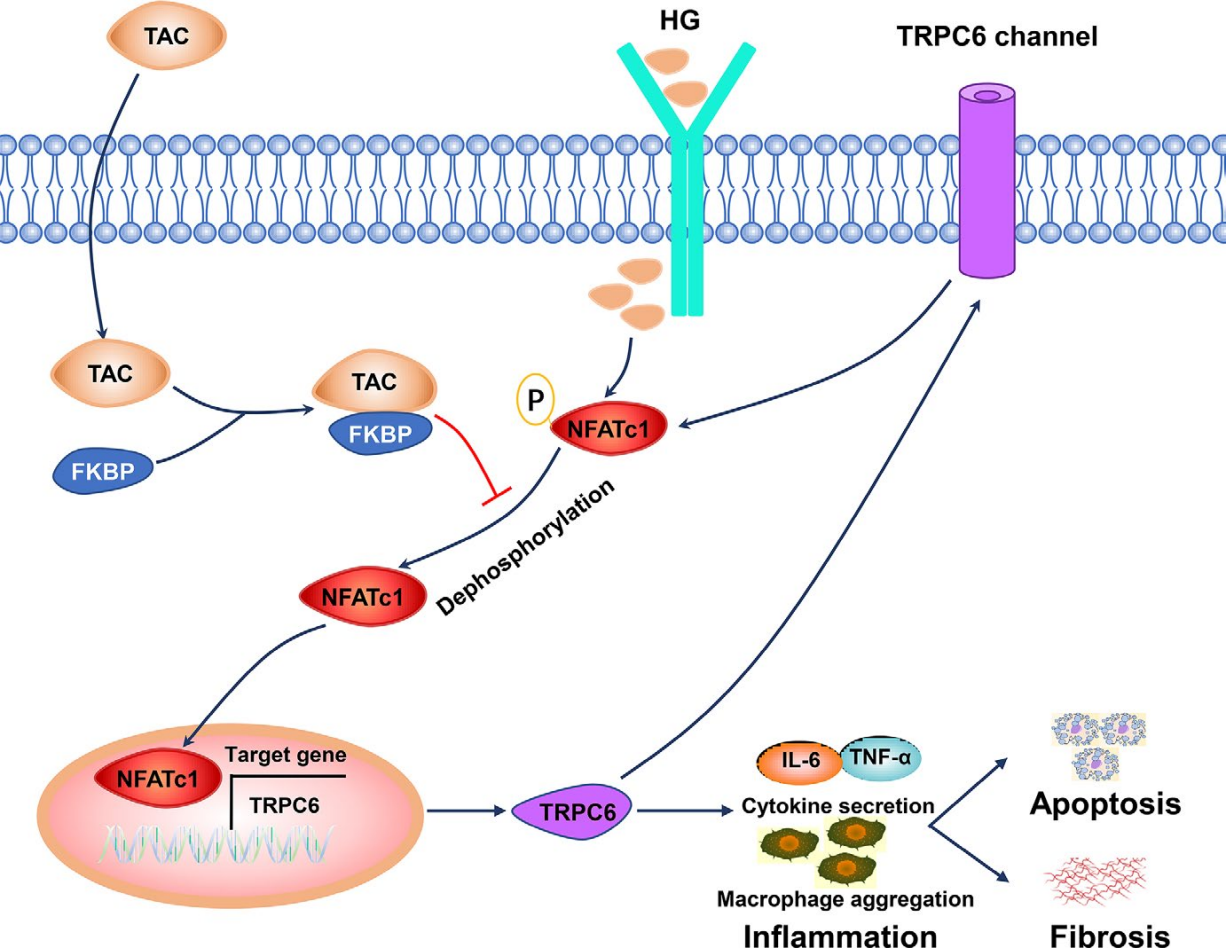
Diagram of the potential mechanism by which tacrolimus (TAC) protects against tubulointerstitial inflammation and fibrosis in diabetic nephropathy (DN). Nuclear factor of activated T cell 1 (NFATc1) is dephosphorylated and translocates to the nucleus in tubular cells in hyperglycaemia conditions. NFAT targets downstream genes, such as transient receptor potential channel 6 (TRPC6), and causes macrophage aggregation and inflammatory cytokine release, eventually leading to tubulointerstitial inflammation and fibrosis. Encouragingly, treatment with TAC significantly inhibits NFATc1 dephosphorylation and nuclear translocation and reduces the transcriptional activation of TRPC6, which ameliorates macrophage infiltration and inflammatory cytokine expression. On the other hand, TRPC6 also induces NFATc1 dephosphorylation and nuclear translocation. The positive feedback loop between NFATc1 and TRPC6 contributes to tubulointerstitial inflammation and injury in DN with TAC treatment

In conclusion, this study delineated that TAC has a potential effect on the amelioration of tubuloinflammation and fibrosis, partially through NFATc1/TRPC6 (Figure 8), which provides more evidence for the renoprotective effects of TAC under hyperglycaemia.

## CONFLICTS OF INTEREST

The authors declare that there are no conflicts of interest.

## AUTHOR CONTRIBUTION

**Shumin Zhang:** Conceptualization (lead); Data curation (lead); Formal analysis (lead); Investigation (lead); Methodology (lead); Project administration (lead); Writing‐original draft (lead); Writing‐review & editing (lead). **Huafen Wang:** Data curation (equal); Formal analysis (equal); Investigation (equal). **Yifei Liu:** Data curation (equal); Formal analysis (equal); Investigation (equal). **Wenxia Yang:** Data curation (equal); Formal analysis (equal); Methodology (equal). **Jialu Liu:** Data curation (equal); Investigation (equal). **Yuzhang Han:** Data curation (equal); Investigation (equal). **Yu Liu:** Formal analysis (equal); Resources (equal); Software (equal); Supervision (equal); Validation (equal); Visualization (equal). **Fuyou Liu:** Funding acquisition (equal); Project administration (equal); Resources (equal); Software (equal); Supervision (equal); Validation (equal); Visualization (equal). **Lin Sun:** Formal analysis (equal); Resources (equal); Software (equal); Supervision (equal); Visualization (equal). **li xiao:** Conceptualization (lead); Formal analysis (lead); Funding acquisition (lead); Methodology (lead); Project administration (lead); Resources (lead); Supervision (lead); Validation (lead); Visualization (lead); Writing‐original draft (lead); Writing‐review & editing (lead).

## Data Availability

The data included in this study are available from the corresponding author upon reasonable request.
